# A method for calculating land degradation neutrality

**DOI:** 10.1016/j.mex.2020.100969

**Published:** 2020-06-19

**Authors:** Sabit Erşahin

**Affiliations:** Department of Forest Engineering, School of Forestry, Çankırı Karatekin University, 18200 Çankırı, Turkey

**Keywords:** Climate change, Land governance quality, Land cover quality, Land quality, Proxy parameter, Soil quality, Water quality

## Abstract

Land degradation neutrality (LDN) has been introduced by United Nations Convention to Combat Desertification (UNCCD). Studies of LDN has been encouraged worldwide by UNCCD to compact land degradation. The LDN aims to maintain or even improve the land quality over time and therefore, it envisages quantifying the balance between the gains and losses within a given land type and scale. In this regard, a mathematical model was developed to calculate and redress land degradation. The calculations showed that the model is stable within the values of quality indices (1 and 2) and correlation coefficients (0 and 1).

• The model calculates a proxy parameter as a representative of land quality using a set of land quality indices and correlation coefficients between those indices.
• The model compares the values for proxy variable for initial and degraded conditions, and calculates the gains needed to equalize the two values.
• The model is independent of scale and it is easy to use.

## Graphical abstract

**Figure:**
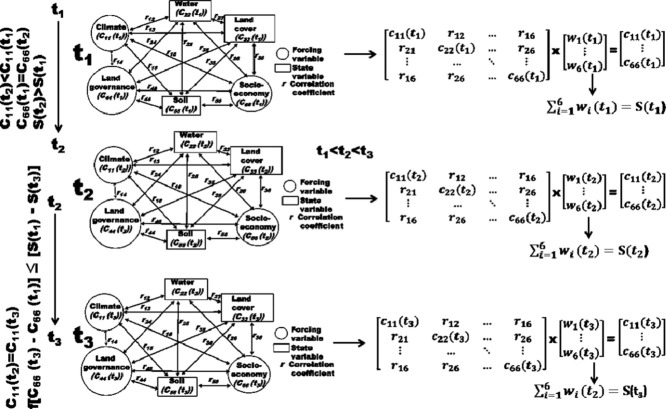

**Table:** Specifications Table

Subject Area:	Environmental Science
More specific subject area:	Land degradation
Method name:	Modeling of land degradation neutrality
Name and reference of original method:	Not applicable
Resource availability:	Not applicable

## Background

Land refers to combined elements of climate, hydrology, soil, vegetation, fauna, and landforms [1]. “Land quality is a multi-faceted term, which encompasses productivity, functions, ecosystem services and their resilience, regeneration capacity, soil and ecosystem health, land potential, etc., individually and in combination” [2]. Land quality indicators are widely used in evaluating the vulnerability of a land to degradation [3]. Land quality indicators should encompass environmental, economic, and social indicators [2].

The concept of land degradation neutrality (LDN) was introduced by the United Nations Convention to Combat Desertification (UNCCD) [2]. The objective of LDN is to maintain or even improve the amount of ecologically healthy and productive land resources over time [4]. “The concept was raised to galvanize effort around a concrete target of “no net loss” and it aims to maintain the world's resource of healthy and productive land through a dual-pronged approach of measures to avoid or reduce land degradation, combined with measures to reverse existing degradation, such that losses are balanced by gains” [4]. Therefore, monitoring LDN envisages quantifying the balance between the gains and losses within a given land type and scale [2,4]. The scope of conceptual framework for LDN should comprise socioeconomic as well as biophysical aspects [4].

## Method details

**Fig. 1:**
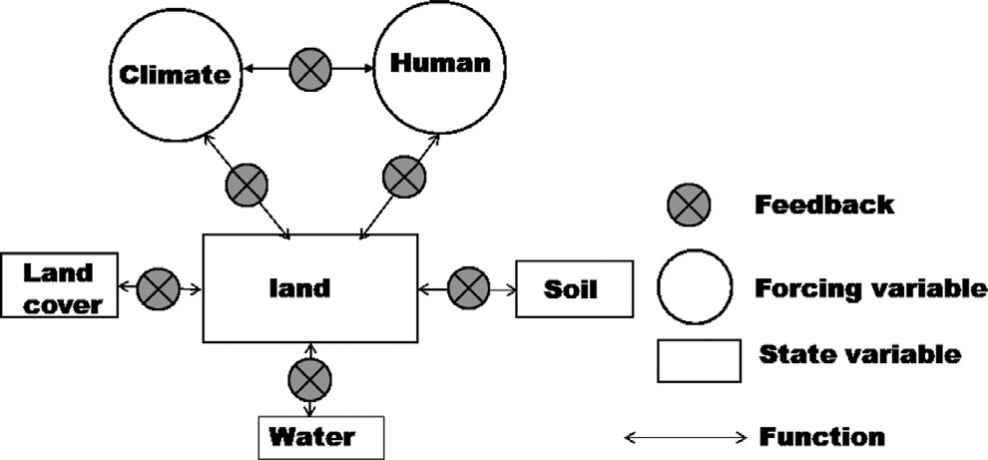
A Conceptual diagram showing land quality indices and interactions between the indices (number of indices may be case-sensitive).

**Fig. 2:**
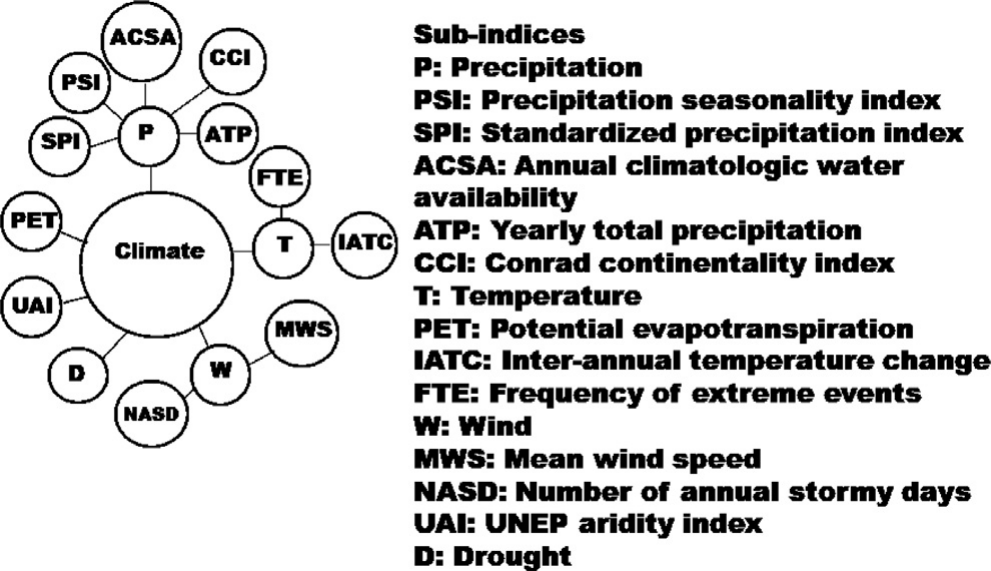
Sub-indices of climate quality indicator (forcing variable).

**Fig. 3:**
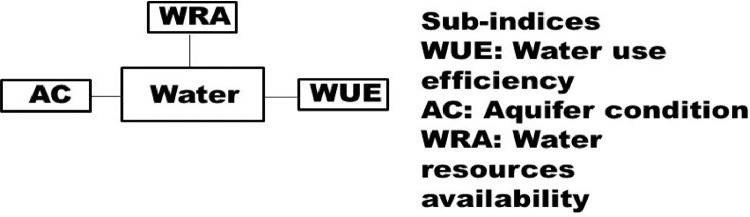
Sub-indices of water quality indicator (state variable).

**Fig. 4:**
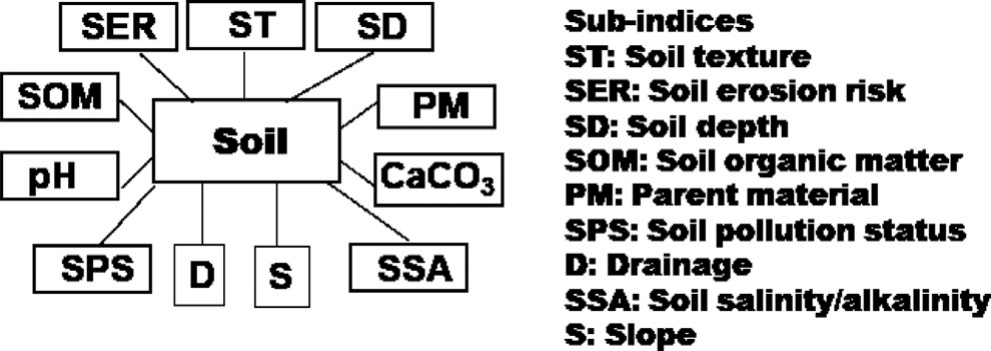
Sub-indices of soil quality indicator (state variable).

**Fig. 5:**
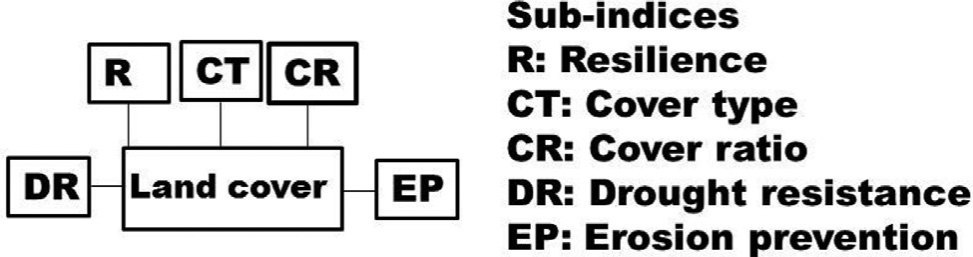
Sub-indices of land cover quality (state variable).

**Fig. 6:**
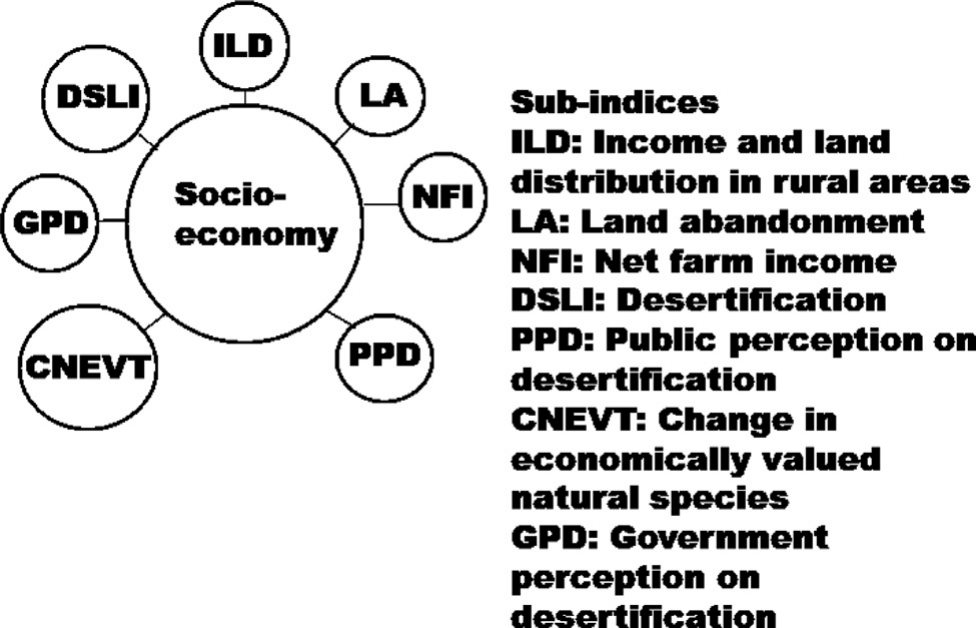
Sub-indices for socio-economy quality indicator (forcing variable).

**Fig. 7:**
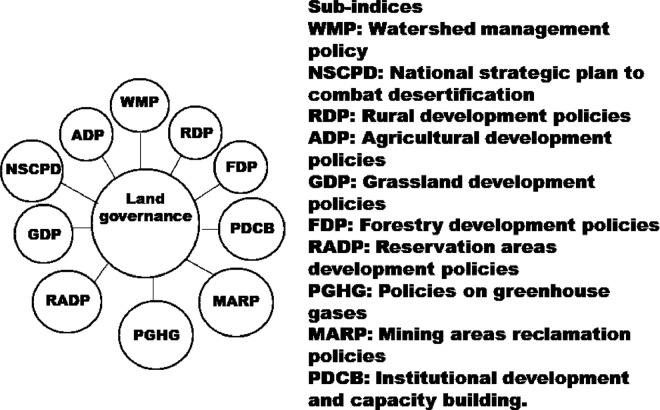
Sub-indices for land governance quality indicator (forcing variable).

**Fig. 8:**
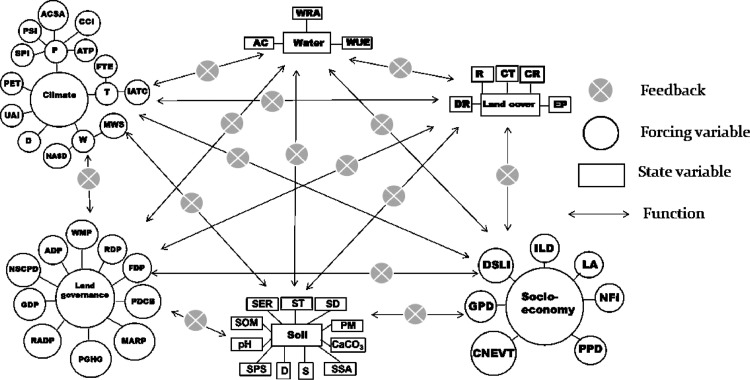
Conceptual diagram showing the land quality indices and their interaction.

Land is a dynamic system, and it constitutes several state and forcing variables (indicators) (Figs. 1 and 2). The quality of a land is simply a combined function of the quality of its components and internal dynamics of the system, which is controlled by multiple interaction among system components. Therefore, those variables may be considered as land quality indices. Upon a perturbation acts over a system at equilibrium, the system reacts, and a new equilibrium is established. A negative impact will result in land degradation (decreased land quality), while an opposite will result in land restoration (increased land quality). The losses in land quality due to negative impact of a stressor may be neutralized by equivalent gains achieved by improved land management. Therefore, in land degradation perspective, for example, losses due to decreased climate quality may be neutralized by gains in land governance quality (Figs. 1,7 and 8). The land system (framework) is illustrated in Fig. 1 and key elements (indices) for land quality are given in Figs. 1 through 8. In the Figs. 1–8, forcing variable refers to external variable of the system, which acts over state variables.

The indices in the Fig. 1 may be disaggregated into their sub-indices as shown in Fig. 2, Fig. 3, Fig. 4, Fig. 5, Fig. 6, Fig. 7.

There are many sub-indices for climate quality indicator (CQI) (Fig. 2). The number of sub-indices may differ depending on data availability and climate type. Some of the sub-indices (e.g., PSI) are function of the others. Fig. 3 shows water quality indicator (WQI) and its sub-indices. Similarly to CQI, the number of sub-indices for WQI may differ depending on data availability, scale of study, and conditions of the land, for example, presence or absence of an aquifer.

Fig. 4 shows sub-indices listed under soil quality indicator (SQI). Similarly, several sub-indices are listed under the land (vegetation) cover quality indicator (LCQI) (Fig. 5). The socio-economy quality indicator (SEQI) (Fig. 6) and land governance quality indicator (LGQI) (Fig. 7) are two indices representing human.

The components shown in Fig. 2–7 can be linked as depicted in Fig. 8.

### Computations

Numerical values are needed for CQI, SQI, LCQI, WQI, SEQI, and LGQI (Fig. 8) to calculate system's quality. The numerical values for each of those indices in Fig. 8 may be calculated similarly to the procedure developed for DIS4ME [5], where the score for an indicator's quality is calculated as follows:

(1) $$QI_{i}={\left(SI_{1}\;x\;SI_{2}\;x........SI_{n}\right)}^{1/n};\;1.0\leq SI_{i}\leq 2.0$$

Where, *QI_i_* is the quality of indicator *i*, and SI_1_, SI_2_, and SI_n_ are the scores given to the quality of sub-indices *1, 2*, and *n*, respectively. For example, if the indicator *I****_i_*** represents soil quality; sub-indicator SI_1_ may be the score for soil texture, SI_2_ for soil organic matter content, and so on. A greater *QI_i_* indicates a better quality for indicator *i*. To exemplify how to use Eq. (1), let sub-indices for LCQI in Fig. 5 be 1.4 for resilience (*R*), 1.5 for cover type (*CT*), 1.3 for cover ratio (*CR*), 1.4 for drought resistance (*DR.*), and 1.6 for erosion prevention (*EP*). Then score for *LCQI* is calculated as follows:

$$LCQI={\left(RxCTxCRxDRxEP\right)}^{1/5}=\;{\left(1.4x1.5x1.3x1.4x1.6\right)}^{1/5}=1.43$$

Scores for other components in the Fig. 8 can be calculated in the same way.

The correlation coefficients between land quality indices in the Fig. 8 should be obtained experimentally, which is a challenging task to accomplish since it needs substantial amount of research. For simplicity, let hypothetical values of correlation coefficients among land quality indices represent the functions and feedbacks among those indices.

The conceptual diagram depicted in Fig. 8 can be defined in a matrix form. It is noteworthy that the number of components may differ depending on scale of the study and land type. In this study, to simplify the example, the land quality attributes were limited to five: CQI, SQI, LCQI, SEQI, and LGQI (Table 1).

**Table 1:** hypothetical values of land quality indices and correlation coefficients between the indices.

	CQI	SQI	LCQI	SEQI	LGQI
CQI	1.4	0.7	0.6	0.7	0.8
SQI	0.7	1.5	0.7	0.7	0.6
LCQI	0.6	0.6	1.5	0.6	0.6
SEQI	0.7	0.7	0.6	1.6	0.7
LGQI	0.8	0.6	0.6	0.7	1.7

CQI: Climate quality index, SQI: Soil quality index, LCQI: Land cover quality index, SEQI: Socio-economy quality index, LGQI: Land governance quality index.

The matrix form of the system in Fig.8 may be defined by Eq. (2).

(2) $$\bar{C}x\bar{W}=\bar{Z}$$

Where,

$$\bar{C}=\left[\begin{array}{cccc} c_{11} & r_{12} & \ldots & r_{1n}\\ r_{21} & c_{22} & \ldots & r_{2n}\\ \vdots & \ldots & \ddots & \vdots \\ r_{1n} & r_{2n} & \ldots & c_{nn} \end{array}\right],\bar{W}=\left[\begin{array}{c} w_{1}\\ \vdots \\ w_{n} \end{array}\right],\;\text{and}\;\bar{Z}=\left[\begin{array}{c} c_{11}\\ \vdots \\ c_{nn} \end{array}\right]$$

In $\bar{C},\;\bar{W}$, and $\bar{Z}$;  *r*_11_ to  *r*_nn_ are correlation coefficients between land quality indices of CQI, SQI, LCQI, SEQI, and LGQI; *c*_11_ to *c*_nn_ are values for land quality indices, and *w*_i_ to *w*_n_ are unknown weights, which are calculated using the elements of $\bar{Z}$−vector and $\bar{C}$ − matrix.

The system of equations in (Eq. (2)) is solved for the weights (*w*_s_) by elementary linear algebra [6]:

(3) $${\bar{C}}^{-1}\;x\;\bar{Z}=\;\bar{W}$$

A perturbation generated in the $\bar{C}$−space forces all the elements in ${\bar{C}}^{-1}\;$to change, resulting in elements of $\bar{W}$−space to alter due to enforced new equilibrium. To illustrate, let one of those indices in $\bar{C}$−space change. The impact due to perturbation resulted from the change will be transmitted to $\bar{W}$ space through ${\bar{C}}^{-1}\times \bar{Z}$. The extent of total change in $\bar{W}$−space will be a combined function of magnitude of decrease in values of indices plus the internal dynamic of the system, which is controlled by the correlation coefficients among the system's components (indices). In land degradation perspective, a decrease in CQI will result in increase in ∑*w_i_* (sum of weights (*SW*)), suggesting land degradation. Difference between initial and final values of *SW* may indicate the extent of land degradation caused by the perturbation due to decreased CQI. To neutralize the degradation caused on the land quality by decreased CQI, value for LGQI can be increased until the difference between initial (∑*w_ii_*) and final (∑*w_if_*) values for ∑*w_i_* becomes negligible. Therefore,

(4) $$\;\sum w_{if}\;-\sum w_{ii}\;\leq 0\;\text{or}\;SW_{f\;}-SW_{i}\leq 0$$

Where, ∑*w_ii_* and ∑*w_if_* or *SW*_f_ and *SW*_i_ are sum of initial and final weights, respectively. Thus, the parameter *SW* can be used as a proxy parameter in monitoring LDN since it merges the indicators and multiple interaction among the indicators.

### Assumptions

The interactions among the system's components (quality indices) are controlled by corresponding correlation coefficients and all correlations are linear.

### Example

The application of the method is exemplified using hypothetical data. Table 1 shows hypothetical values for CQI, SQI, VCQI, SEQI, and LGQI and the hypothetical values for correlation coefficients between the quality indices. In Table 1, on-diagonal entries from left top to right bottom are hypothetical values for quality indices and off-diagonal entries are hypothetical values for correlation coefficients between those indices.

Using the data in the Table 1 with the system given in Eq. (2), the following system of equations is resulted:

(5) $$\bar{C}=\;\left[\begin{array}{ccccc} 1.5 & 0.7 & 0.6 & 0.7 & 0.5\\ 0.7 & 1.5 & 0.7 & 0.7 & 0.6\\ 0.6 & 0.6 & 1.5 & 0.6 & 0.6\\ 0.7 & 0.7 & 0.6 & 1.6 & 0.7\\ 0.5 & 0.6 & 0.6 & 0.7 & 1.4 \end{array}\right]x\left[\begin{array}{c} w_{CQI}\\ w_{SQI}\\ w_{LCQI}\\ w_{SEQI}\\ w_{LGQI} \end{array}\right]=\left[\begin{array}{c} 1.5\\ 1.5\\ 1.5\\ 1.6\\ 1.4 \end{array}\right]$$

where, *w_CQI_* through *w_LGQI_* are weights for climate, soil, land cover, socico-economy, and land governance quality indices, respectively.

By Eq. (3), the weights for indices are calculated as follows:

$$w=\left[\begin{array}{c} 0.41\\ 0.27\\ 0.36\\ 0.43\\ 0.37 \end{array}\right],\sum w_{i}=SW=1.86$$

*To illustrate how land degradation is quantified by a proxy variable (SW)*

1. Let the following system of equations represent a land initially at equilibrium (See Table 1 and Eq. (2)).
  $$\bar{C}=\;\left[\begin{array}{ccccc} 1.5 & 0.7 & 0.6 & 0.7 & 0.5\\ 0.7 & 1.5 & 0.7 & 0.7 & 0.6\\ 0.6 & 0.6 & 1.5 & 0.6 & 0.6\\ 0.7 & 0.7 & 0.6 & 1.6 & 0.7\\ 0.5 & 0.6 & 0.6 & 0.7 & 1.4 \end{array}\right]x\left[\begin{array}{c} w_{CQI}\\ w_{SQI}\\ w_{LCQI}\\ w_{SEQI}\\ w_{LGQI} \end{array}\right]=\left[\begin{array}{c} 1.5\\ 1.5\\ 1.5\\ 1.6\\ 1.4 \end{array}\right]$$

By Eq. (3):

$$w=\left[\begin{array}{c} 0.41\\ 0.27\\ 0.36\\ 0.43\\ 0.37 \end{array}\right],\sum w_{ii}=SW_{i}=1.84$$

2. Let CQI in above system decrease from 1.5 to 1.3 (notice the figures typed in bold) resulted from climate change.
  $$\bar{C}=\;\left[\begin{array}{ccccc} 1.3 & 0.7 & 0.6 & 0.7 & 0.5\\ 0.7 & 1.5 & 0.7 & 0.7 & 0.6\\ 0.6 & 0.6 & 1.5 & 0.6 & 0.6\\ 0.7 & 0.7 & 0.6 & 1.6 & 0.7\\ 0.5 & 0.6 & 0.6 & 0.7 & 1.4 \end{array}\right]x\left[\begin{array}{c} w_{CQI}\\ w_{SQI}\\ w_{VCQI}\\ w_{SEQI}\\ w_{LGQI} \end{array}\right]=\left[\begin{array}{c} 1.5\\ 1.5\\ 1.5\\ 1.6\\ 1.4 \end{array}\right],$$
  $$w=\left[\begin{array}{c} 0.34\\ 0.29\\ 0.38\\ 0.44\\ 0.37 \end{array}\right],\sum w_{if}=SW_{f}=1.90$$

Difference is 1.84–0.90 = −0.06, which indicates land degradation due to decreased CQI.

3. Let CQI increase from 1.5 to 1.7.
  $$\bar{C}=\;\left[\begin{array}{ccccc} 1.7 & 0.7 & 0.6 & 0.7 & 0.5\\ 0.7 & 1.5 & 0.7 & 0.7 & 0.6\\ 0.6 & 0.6 & 1.5 & 0.6 & 0.6\\ 0.7 & 0.7 & 0.6 & 1.6 & 0.7\\ 0.5 & 0.6 & 0.6 & 0.7 & 1.4 \end{array}\right]x\left[\begin{array}{c} w_{CQI}\\ w_{SQI}\\ w_{VCQI}\\ w_{SEQI}\\ w_{LGQI} \end{array}\right]=\left[\begin{array}{c} 1.5\\ 1.5\\ 1.5\\ 1.6\\ 1.4 \end{array}\right],$$
  $$w=\left[\begin{array}{c} 0.34\\ 0.29\\ 0.38\\ 0.44\\ 0.37 \end{array}\right],\sum w_{if}=SW_{f}=1.80$$

Difference is 1.86–1.80 = 0.06, which indicates land restoration due to increase in CQI.

The above calculations show that total change in the sum of the weights (*SW*) indicates total decrease or increase in systems quality as a response to changes in CQI, suggesting that change in the parameter *SW* may be used as a quantitative measure of extent of land degradation or restoration.

**Fig. 9:**
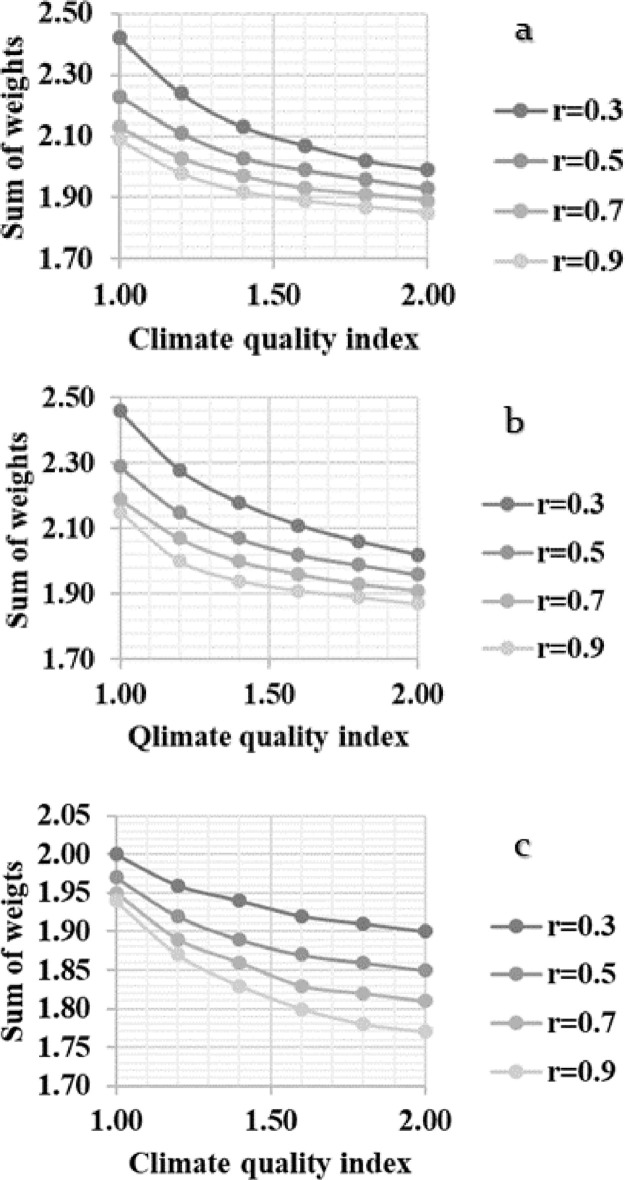
Response of sum of weights (*SW*) to decrease in climate quality index at different values of 229 correlation coefficients between a) climate quality index and land governance quality index, b) climate 230 quality index and land cover quality index, and c) soil quality index and land cover quality index.

Fig. 9 shows sensitivity of *SW* against gradually decreasing CQI in below initial system (notice the bold typed figures). Fig. 9 depicts that curves representing response of *SW* to gradually decreased values of CQI shifts upward as a response to decreased r-values, representing association between CQI and LGQI, CQI and LCQI, and LCQI and SQI in Figs.9a, 9b, and 9c, respectively. Fig. 9 further depicts that both of upward shifting and shape of the curves are specific to correlation coefficients between the quality indices.

$\bar{C}=\left[\begin{array}{ccccc} 2.0 & 0.7 & 0.6 & 0.7 & 0.5\\ 0.7 & 1.5 & 0.7 & 0.7 & 0.6\\ 0.6 & 0.6 & 1.5 & 0.6 & 0.6\\ 0.7 & 0.7 & 0.6 & 1.6 & 0.7\\ 0.5 & 0.6 & 0.6 & 0.7 & 1.4 \end{array}\right]x\left[\begin{array}{c} w_{CQI}\\ w_{SQI}\\ w_{VCQI}\\ w_{SEQI}\\ w_{LGQI} \end{array}\right]=\left[\begin{array}{c} 2.0\\ 1.5\\ 1.5\\ 1.6\\ 1.4 \end{array}\right]$, which results in following weights

$$w=\left[\begin{array}{c} 0.34\\ 0.29\\ 0.38\\ 0.44\\ 0.37 \end{array}\right],SW_{i}=1.89$$

### To illustrate how to neutralize land degradation

1. Consider the below initial system.
  $$\bar{C}=\;\left[\begin{array}{ccccc} 1.5 & 0.7 & 0.6 & 0.7 & 0.5\\ 0.7 & 1.5 & 0.7 & 0.7 & 0.6\\ 0.6 & 0.6 & 1.5 & 0.6 & 0.6\\ 0.7 & 0.7 & 0.6 & 1.6 & 0.7\\ 0.5 & 0.6 & 0.6 & 0.7 & 1.4 \end{array}\right]x\left[\begin{array}{c} w_{CQI}\\ w_{SQI}\\ w_{VCQI}\\ w_{SEQI}\\ w_{LGQI} \end{array}\right]=\left[\begin{array}{c} 1.5\\ 1.5\\ 1.5\\ 1.6\\ 1.4 \end{array}\right]$$
  $$w=\left[\begin{array}{c} 0.42\\ 0.23\\ 0.38\\ 0.39\\ 0.46 \end{array}\right],SW_{i}=1.84$$
2. Let the value for CQI decrease from 1.5 to 1.3 as shown below. The decrease results in *SW*_i_*- SW*_f_ *=* −0.06, which suggests land degradation.
  $$\bar{C}=\;\left[\begin{array}{ccccc} 1.3 & 0.7 & 0.6 & 0.7 & 0.5\\ 0.7 & 1.5 & 0.7 & 0.7 & 0.6\\ 0.6 & 0.6 & 1.5 & 0.6 & 0.6\\ 0.7 & 0.7 & 0.6 & 1.6 & 0.7\\ 0.5 & 0.6 & 0.6 & 0.7 & 1.4 \end{array}\right]x\left[\begin{array}{c} w_{1}\\ w_{2}\\ w_{3}\\ w_{4}\\ w_{5} \end{array}\right]=\left[\begin{array}{c} 1.5\\ 1.5\\ 1.5\\ 1.6\\ 1.4 \end{array}\right]$$
  $$w=\left[\begin{array}{c} 0.42\\ 0.23\\ 0.38\\ 0.39\\ 0.46 \end{array}\right],SW_{f}=1.90$$
3. The value for LGQI should be adjusted from 1.4 approximately to 1.70 to decrease *SW* from 1.90 to 1.84 as shown below.
  $$\bar{C}=\;\left[\begin{array}{ccccc} 1.3 & 0.7 & 0.6 & 0.7 & 0.5\\ 0.7 & 1.5 & 0.7 & 0.7 & 0.6\\ 0.6 & 0.6 & 1.5 & 0.6 & 0.6\\ 0.7 & 0.7 & 0.6 & 1.6 & 0.7\\ 0.5 & 0.6 & 0.6 & 0.7 & 1.70 \end{array}\right]x\left[\begin{array}{c} w_{1}\\ w_{2}\\ w_{3}\\ w_{4}\\ w_{5} \end{array}\right]=\left[\begin{array}{c} 1.5\\ 1.5\\ 1.5\\ 1.6\\ 1.4 \end{array}\right]$$
  $$w=\left[\begin{array}{c} 0.54\\ 0.24\\ 0.40\\ 0.43\\ 0.25 \end{array}\right],SW_{f}=SW_{i}=1.84$$

The neutralizing efficacy of a gain via increased LGQI depends partly on the interaction between the land system components. To illustrate, let correlation coefficient between CQI and LGQI be 0.6 instead of 0.5. In this case, the below calculations show that increasing LGQI from 1.40 to 1.47 is enough to neutralize the land degradation.

$$\bar{C}=\;\left[\begin{array}{ccccc} 1.3 & 0.7 & 0.6 & 0.7 & 0.6\\ 0.7 & 1.5 & 0.7 & 0.7 & 0.6\\ 0.6 & 0.6 & 1.5 & 0.6 & 0.6\\ 0.7 & 0.7 & 0.6 & 1.6 & 0.7\\ 0.5 & 0.6 & 0.6 & 0.7 & 1.47 \end{array}\right]x\left[\begin{array}{c} w_{1}\\ w_{2}\\ w_{3}\\ w_{4}\\ w_{5} \end{array}\right]=\left[\begin{array}{c} 1.5\\ 1.5\\ 1.5\\ 1.6\\ 1.4 \end{array}\right]$$

$$w=\left[\begin{array}{c} 0.50\\ 0.25\\ 0.40\\ 0.43\\ 0.28 \end{array}\right],SW_{f}=1.84$$

## Conclusions

“LDN has two linked dimensions: i) reducing the rate of degradation of non-degraded-land; and ii) increasing the rate of restoration of degraded land” [2]. The model and framework developed in this study may serve to achieve both of those dimensions. For example, the scenarios of land degradation can be tested hypothetically to anticipate gains needed to counterbalance likely future losses in land quality. It should be noted that LDN does not coincide return of the degraded land to its pristine conditions, but it means a new state of land, which is equivalent to the original condition in land quality. The model developed in this study allows estimation of viability and effectiveness of the offsetting mechanisms and interventions to be applied to counterbalance losses in the land quality.

The model is simple and easy to conduct, and it is robust and flexible as well; it provides pragmatic prognosis within different spatial and temporal limits; it can be basis for the setting the functions, which control inter-indicator relations, for selection of the most proper indicators and dynamics of indicators and metrics for LDN across scales. The model can be further improved to account for co-benefits and negative side effects of restoration activities in counterbalancing interventions.

The two hypotheses are tested using hypothetical data. The sum of weights (∑*w_i_* ≡ *SW*) for quality indices responded consistently to gradual changes in land quality indicators. The calculations showed that the system is stable with the values between the ranges of quality indices (1 and 2) and correlation coefficients (0 and 1). The model is highly flexible and robust that it can be applied case and scale specifically. Also, the model may be used as an education and research tool to rapidly evaluate and understand the land degradation neutrality scenarios under different combination of land quality indices and functional relationships between those indices. However, the model cannot yield realistic results unless it is calibrated and verified by real data. Therefore, research is needed to: 1. Define proper land quality indices and sub-indices across different scales and land types, and more importantly 2. Develop case- and scale-specific correlation coefficients, describing inter-relations among systems’ components, which controls the internal dynamics of the land system.

## Declaration of competing interest

The author declares that he has no known competing financial interests or personal relationships that could have appeared to influence the work reported in this paper.
